# A Textbook of Gynæcological Surgery

**Published:** 1911-09

**Authors:** 


**A Textbook of Gynæcological Surgery.** By Comyns Berkeley, M.A., M.D., B.C., and Victor Bonney, M.S., M.D., F.R.C.S. Pp. xi, 720. London: Cassell & Company Ltd. 1911.

This is a new and very handsome work. Its illustrations are throughout extremely clear and of ample number, whilst the coloured plates are of a very high order of artistic merit. The text represents the teaching of the Middlesex Hospital. A detailed account is first given of general surgical technique, which we regard as rather redundant in a book of this description. The method of skin preparation is the old-fashioned routine, involving many changes of lotion. The iodine method is briefly mentioned, and the reader is allowed to take his choice.

The section dealing with perineal operations suffers from an excessive elaboration of detail, whilst the important role of the levatores ani is barely mentioned, and these structures do not appear in one of the eighteen pictures which illustrate this subject.

There is a good account of the different methods of hysterectomy, with a discussion of their several merits. The wealth of illustrations accompanying the account of these operations makes it extremely clear. The book concludes with a very careful account of the after-treatment of operations, post-operative complications, and results.

